# Rheological Optimization of Reduced-Sugar Ice Cream Enriched with Inulin and Carob Flour

**DOI:** 10.3390/foods15193362

**Published:** 2026-09-22

**Authors:** Teresa Stefania Dell’Endice, Giusy Rita Caponio, Anna Maria Giudetti, Davide De Angelis, Vito Michele Paradiso

**Affiliations:** 1 Department of Clinical and Experimental Medicine, University of Foggia, Via Napoli 121, 71122 Foggia, Italy; stefania.dellendice@unifg.it; 2 Department of Biological and Environmental Sciences and Technologies, University of Salento, S. P. 6 Lecce-Monteroni, 73100 Lecce, Italy; anna.giudetti@unisalento.it (A.M.G.); vito.paradiso@unisalento.it (V.M.P.); 3 Department of Bioscience, Biotechnology and Environment, University of Bari Aldo Moro, Via Orabona 4, 70125 Bari, Italy; giusy.caponio@uniba.it; 4 Department of Soil, Plant and Food Science (DISSPA), University of Bari Aldo Moro, Via Amendola, 165/a, 70126 Bari, Italy

**Keywords:** ice cream reformulation, rheological properties, sugar reduction, mixture design, inulin

## Abstract

Sugar reduction is technologically challenging in ice cream because sugars contribute not only to sweetness but also to rheological properties. A D-optimal mixture design was applied to optimize the formulation of reduced-sugar ice cream using inulin and carob flour as functional ingredients. The effects of sucrose, dextrose, inulin, and carob flour on viscosity, consistency index, flow behavior index, and thixotropy were investigated. All formulations exhibited pseudoplastic behavior, although marked differences in rheological properties were observed within the experimental domain. Sucrose increased viscosity and consistency, whereas dextrose reduced both parameters. Inulin and carob flour exerted a pronounced thickening effect due to their water-binding capacity. Based on the rheological targets defined using a commercial reference ice cream, an optimized formulation was identified and experimentally validated. The optimized sample showed a broadly comparable rheological profile to the commercial reference, although some discrepancies between predicted and experimental values were observed. Compared with both the experimental and commercial controls, the optimized formulation showed lower sucrose and glucose contents, corresponding a reduction of approximately 71% in glucose and sucrose. Total dietary fiber (calculated value) reached 11.59 g 100 g^−1^, allowing the product to be potentially classified as “high in fiber” with a lower energy value. Sensory evaluation revealed a significant reduction in perceived sweetness (−25%) compared to the control formulation. Inulin and carob flour can be effectively used to develop reduced-sugar ice cream with improved nutritional quality while maintaining properties in agreement with the formulation targets.

## 1. Introduction

Excessive sugar consumption has been associated with chronic metabolic diseases such as diabetes and obesity, poor dental health, and increased cardiovascular risk [1,2]. Accordingly, both the U.S. Department of Agriculture (USDA) and the World Health Organization (WHO) recommend limiting added sugars to 6–10% of total daily energy intake [3]. However, the sugar intake frequently exceeds these levels, reaching 12–15% of total energy in the United States and several European countries [4,5]. In response, sugar reduction reformulation strategies have been explored for several food categories, including ice cream [6,7].

However, reformulating ice cream to reduce sugar content is technologically challenging because sugars carry out several functions that extend far beyond sweetness [6,8,9]. Specifically, in ice cream and other dispersed food systems, sucrose and other related sugars influence several physicochemical, rheological, and structural properties [8]. Sugars reduce the freezing point of ice cream and modulate the ice crystallization during preparation, having direct consequences on hardness, melting resistance, and sensory perceptions [8,9,10,11]. For these reasons, reducing or partially replacing sucrose typically leads to ice cream that is harder, less stable, and more prone to structural collapse unless additional hydrocolloids, fibers, or polyols are incorporated to compensate the reduced functionality [9,12]. Rheological properties are particularly relevant for characterizing the effects of formulation changes, as they describe the flow and deformation behavior of the ice cream matrix and are influenced by the composition and interactions of its components. Viscosity and consistency characterize resistance to flow, whereas the flow behavior index describes the degree of shear-thinning and thixotropy reflects structural changes induced by shear. These parameters are therefore useful for assessing the effects of sugar replacement and functional ingredients on ice cream rheology [13,14].

Consequently, a key challenge is to identify combinations of functional ingredients that can reproduce the rheological profile of conventional ice cream while simultaneously improving the nutritional composition. To address this technological gap, a formulation strategy involving the use of Design of Experiments (DoE) for mixtures can be used [15]. This is in line with the growing interest in functional foods, including ice cream [16,17] as a strategy to support the prevention of metabolic diseases [18].

Regarding the ingredients, a growing number of studies have investigated the use of dietary fibers as functional ingredients in frozen desserts. Among these, inulin is a fructan-type plant polysaccharide composed of β-(2 → 1)-linked fructose units, and it is primarily extracted from chicory roots [19] or from artichoke [20]. From a technological perspective, inulin improves ice cream texture by binding water molecules and forming a gel network, positively affecting rheological behavior [21]. It has been shown to act as a sugar and fat replacer, even at low concentrations [13,16,21,22], while also improving nutritional quality by increasing fiber content and providing prebiotic benefits [16].

Carob flour is another ingredient of interest. It is obtained from the pulp of *Ceratonia siliqua* L., a legume widely cultivated in the Mediterranean basin [23]. While carob seeds are commonly used to produce locust bean gum, the pulp is rich in insoluble dietary fiber, sugars (sucrose, glucose, and fructose), and phenolic compounds. It has recently attracted attention as a sustainable ingredient [24]. The nutritional composition of carob flour suggests its possible exploitation as a natural sugar ingredient in ice cream and frozen desserts [23], where it may also play a structural role.

Considering these aspects, in this study, we used a formulation strategy guided by mixture design to optimize the viscosity, consistency, flow behavior index, and thixotropy of ice cream formulations containing functional ingredients (inulin and carob flour) while reducing the content of glucose and sucrose. The objective was to identify a combination of ingredients able to approach predefined rheological targets derived from a commercial reference while reducing the content of glucose and sucrose. The optimized formulation was subsequently experimentally validated, and its nutritional characteristics and sweetness perception were compared with those of the control.

## 2. Materials and Methods

### 2.1. Materials

The ingredients used for ice cream preparation were purchased from local supermarkets, and they included shelf-stable milk (containing 3.6% fat, 3.4% protein, 5% sugar) (Granarolo SpA, Bologna, Italy); skim milk powder (FG Food Srl, Galatone, Italy); whipped cream (containing 35% fat, 2.1% protein) (Parmalat, Milan, Italy); sucrose; inulin from chicory (Daila Srl, Osio Sotto, Italy), with an average degree of polymerization (DP) ~10; carob flour (Rapunzel Naturkost GmbH, Legau, Germany); dextrose (Daila Srl, Osio Sotto, Italy); and a hydrocolloid mix (Comprital SpA, Settala, Italy), which was provided by FG Food (Lecce, Italy) and contained E471 (mono- and diglycerides of fatty acids), sucrose esters of fatty acids (E473), sodium carboxymethyl cellulose (CMC) (E466), and tara gum (E417). A commercial ice cream fiordilatte style (Sammontana SpA, Empoli, Italy) was purchased and used as a commercial reference for the rheological and nutritional properties.

### 2.2. Ice Cream Manufacture

All the ice cream samples were prepared at the Department of Soil, Plant and Food Science, University of Bari Aldo Moro (Italy), using the available equipment. The flow diagram of the ice cream making is shown in Figure 1. For the preparation, cow’s milk was heated at 81 °C for 3 min using an induction cooker (Mod. G10061, G3 Ferrari, Trevidea Group, Rimini, Italy), before adding powdered ingredients, previously sieved with a fine-mesh sieve, and whipped cream, stirring manually and continuously until all components were fully dissolved. The ice cream mixes were then thoroughly blended with an electric mixer (Mod. G20118, G3 Ferrari, Trevidea Group, Rimini, Italy) to achieve complete homogeneity. The mix was refrigerated and transferred to an ice cream maker (Mod. 693, Ariete, Treviso, Italy) where it was creamed for 20 min at −20 °C. After resting overnight at −20 °C, the ice cream was whipped for further 40 min using the ice cream maker to incorporate air and achieve the final texture. The samples were stored at −20 °C until the analysis.

### 2.3. Experimental Design

The experimental domain to study the possible combinations of the main ice cream ingredients was defined according to a 4-component D-Optimal mixture design and a two factor interaction model, as expressed by the following equation:

Y = β1x1 + β2x2 + β3x3 + β4x4 + β12x1x2 + β13x1x3 + β14x1x4 + β23x2x3 + β24x2x4 + β34x3x4(1)

where Y, x, and β are the response variables, the components under investigation, and the coefficients of the model, respectively.

After preliminary trials using a recipe provided by FG Food (Lecce, Italy), the constraints of the model were chosen to optimize the content of carob flour and inulin while minimizing the dextrose and sucrose content in the ice cream, which were (g kg^−1^): sucrose (0 ≤ x1 ≤ 154); dextrose (0 ≤ x2 ≤ 39); inulin (0 ≤ x3 ≤ 193); and carob flour (0 ≤ x4 ≤ 193). The sum of the four ingredients was 193 g/kg. The other ingredients were kept constant and completed the formulation. They consisted of 600 g of full fat milk, 177 g of whipped cream, 25 g of skim milk, and 5 g of a hydrocolloid mix (data are expressed as g kg^−1^). Table 1 reports the formulations of the 15 experimental trials considering the variables under investigation.

### 2.4. Viscosity and Thixotropy Measurements

The viscosity of the ice cream was determined with an oscillatory rheometer (HAAKE MARS iQ Air, Thermo Fisher Scientific, Waltham, MA, USA) equipped with a coaxial cylinder geometry (CCB25 DIN/SS-01200990) under a controlled temperature of 4 °C to guarantee the reproducibility of the results. A rotational shear ramp test was performed by first increasing the shear rate from 10 to 200 s^−1^ before then decreasing the shear rate from 200 to 1 s^−1^. The apparent viscosity was calculated at a shear rate of 50 s^−1^, corresponding to typical shear rate detected in mouth for low-viscosity foods [13]. The data were fitted using the Ostwald–de Waele model:

(2) $$\tau =K\;\;{\dot{\gamma }}^{\;n}$$

where τ = shear stress (Pa), $\dot{\gamma }$ = shear rate (s^−1^), K = consistency index (Pa s^n^), and n = flow behavior index. Chi^2^ and r obtained from the fitting are reported in Supplementary Table S1.

Thixotropy (%) was calculated according to the formula:

Thixotropy (%) = (η − η′)/η × 100(3)

where η and η′ are the viscosities at 50 s^−1^ in the increasing and decreasing shearing steps, respectively.

### 2.5. Sugars Determination and Total Soluble Solids

Sucrose and glucose contents were determined following the method described by [25], with some modification. Specifically, 10 mg of ice cream were dispersed in 10 mL of double distilled water and filtered through 0.22 μm cellulose acetate filters. For the analysis, high-performance liquid chromatography (HPLC) was used (Agilent Technologies, Santa Clara, CA, USA), in combination with a Refractive Index Detector (RID 1260). Separation was carried out with deionized water at a flow rate of 1 mL min^−1^ through a 300 × 7.8 mm cation exchange column (Rezex RCM column, Ca^2+^, 8 μm, Phenomenex, Torrance, CA, USA) at 80 °C. Quantification was made using an external standard calibration curve of sucrose and glucose, and the results were expressed as g 100 g^−1^ of fresh product. Total soluble solids content, expressed as °Brix, was assessed using an Abbe’s refractometer (Carl Zeiss, Oberkochen, Germany). The determinations were carried out three times.

### 2.6. Sensory Analysis

Sensory evaluation of sweetness was carried out by a trained panel (8 women, 5 men aged between 24 and 60 years old). The research followed the protocols designed to protect the rights, privacy, and welfare of participants, who provided written consent to take part in the sensory analysis. Participation in the study was voluntary, and panelists could withdraw from the test at any time. The selected panelists were in good health, had no known allergies to the components, and were informed before the analysis about the purpose and procedures of the study. Participants were assured of the confidentiality of their data. The ice cream samples were stored at −20 °C and tempered at room temperature for 4 min prior to serving. Approximately 10 g of each ice cream sample was served in white plastic containers identified with randomized codes corresponding to the sample abbreviations. A 5 min interval was maintained between tastings, during which participants rinsed their mouths with water to minimize carryover effects. Sample presentation order was randomized among participants and across replicates. The sweetness perception was evaluated on a scale from 0 (absent) to 9 (very intense, i.e., saturated water with sugar).

### 2.7. Statistical Analysis

The experimental design data were fitted to the quadratic model. For each response variable, the coefficient of determination (R^2^), the adjusted coefficient of determination (R^2^_adj_), and the significance of both the overall regression and individual coefficients (*p* ≤ 0.05) were computed using Design-Expert 11 (StatEase Inc., Minneapolis, MN, USA). A lack-of-fit test was not performed because the experimental design did not include independent replicate runs. Residual diagnostics was carried out using Python 3.12, with statsmodels (function statsmodels.stats.diagnostic.het_breuschpagan, v0.15.0). Data of optimized formulation and controls were subjected to one-way ANOVA followed by Tukey’s HSD test (α = 0.05) using Minitab 19 (Minitab Inc., State College, PA, USA). Sweetness scores for the optimized formulation and the experimental control were compared using a paired *t*-test, as each panelist evaluated both samples. The analysis included 13 valid pairs. The normality of the within-panelist differences was assessed using the Shapiro–Wilk test, with *p*-values > 0.10, indicating no significant departure from normality. Statistical significance was set at α = 0.05. The results are reported as mean ± SD of three replicates.

## 3. Results and Discussion

### 3.1. Model Evaluation

Table 2 reports the coefficients of the regression models calculated for the responses, together with their coefficients of determination and significance.

The models for all the responses were statistically significant, as indicated by the high coefficients of determination and low *p*-values, highlighting that the models explained a large proportion of the variability observed within the experimental data. The complete ANOVA results for the four responses are reported in Supplementary Tables S2–S5. Residual diagnostics (Supplementary Figure S1) showed no evident systematic patterns and adequate normality for viscosity, flow behavior index, and thixotropy. For consistency, larger absolute residuals were observed at lower values, including some near-zero or slightly negative predictions, an expected artifact given the ~224-fold range of this response across the design. Additionally, for consistency (K), the Breusch–Pagan test did not indicate significant heteroscedasticity (*p* = 0.61).

When considering the coefficients of the variables, Table 2 shows that, in most of the cases, only the linear terms were generally the most relevant, whereas significant interaction terms were mainly observed for consistency. This indicates that changing the proportion of one ingredient alone has a statistically significant impact on viscosity, flow behavior index and thixotropy, regardless of the interactions with other ingredients.

The analysis of the model coefficients allows the effects of the formulation variables on the rheological responses to be defined. Increasing the sucrose content resulted in higher viscosity and consistency, in agreement with previous findings attributing this effect to its relatively high molecular weight and its ability to structure the unfrozen phase [8]. Conversely, dextrose had a negative effect on the viscosity and consistency of the ice cream, whereas it increased the thixotropic behavior. The lower molecular weight of dextrose compared to sucrose increases the fraction of free water and reduces the structuring of the serum phase, thereby resulting in a less viscous mix [26].

Inulin and carob flour showed positive coefficients, confirming their thickening effect [19,27]. These results confirm the challenges in reducing or replacing sugars in ice cream due to their effect on viscous properties. Nevertheless, the use of DoE as a formulation strategy helps to quantify these effects and to find possible solutions.

To further characterize model performance, the predicted R^2^ (based on the PRESS statistics) and Adequate Precision were also computed (Table 2). While Adequate Precision values (ranging from 9.5 to 17.6) largely exceeded the minimum desirable threshold of four, indicating an adequate signal-to-noise ratio, the predicted R^2^ values were markedly negative for all responses. The negative predicted R^2^ values indicate limited predictive performance under the internal PRESS-based cross-validation procedure. This result should be interpreted considering the relatively small number of experimental runs and the high number of model coefficients, which resulted in only five residual degrees of freedom. Under these conditions, the PRESS-based estimate may be sensitive to individual observations. Therefore, the negative predicted R^2^ values indicate a limitation of the internal cross-validation assessment, but do not, by themselves, establish that the models are unsuitable for formulation development. Similarly, the high R^2^ values should be interpreted as measures of goodness-of-fit rather than as evidence of robust prediction across the entire experimental domain. For this reason, experimental validation with an independent formulation was considered necessary to provide a local assessment of the agreement between predicted and experimental values, as described in Section 3.3.

### 3.2. Rheological Properties of Ice Cream

Table 3 reports the results of the rheological evaluation of the experimental ice cream prepared according to the mixture design. Specifically, the importance of viscosity and consistency must be acknowledged in relation to the technological and sensory properties of ice cream. In fact, viscosity is directly related to the hardness of the ice cream [8,9]. Moreover, viscosity influences the heat transfer during melting, as a higher viscosity increases thermal insulation, thereby slowing the melting rate [8]. This represents a critical factor during serving and consumption. The formulations planned according to the DoE covered a broad range of viscosities, consistencies, flow behaviors, and thixotropic indices, as a wide variability in the rheological behavior of the ice cream mixes was evident. The results show that mixes 1 and 2 were characterized by high viscosity and consistency. These trials correspond to the vertices of the experimental domain, and they were characterized by the maximum levels of inulin and carob flour, respectively, and by the complete absence of added sugars (Table 1). Conversely, formulations containing higher proportions of dextrose tended to exhibit lower viscosity and consistency, in agreement with the negative coefficients in the corresponding models. These formulations also exhibited the highest thixotropy values, indicating a limited ability to maintain viscosity after shear. Similar findings were reported by Caponio et al. [13], who observed increased thixotropic behavior in ice cream enriched with inulin and oat okara compared to the control. This behavior may be attributed to the strong water-binding capacity of inulin and carob flour and to their ability to interact with the aqueous phase, resulting in a more structured network that breaks down under shear but only partially recovers afterward [28]. As indicated by the flow behavior index (n), all formulations exhibited pseudoplastic behavior (n < 1), showing a decrease in viscosity as shear rate increased. This rheological behavior is typical of ice cream systems [28] and other dispersed food matters [29]. The higher n values observed in some formulations, such as trials 5 and 12, were therefore indicative of a comparatively weaker shear-thinning response, whereas the n values observed in trials 1 and 2 indicate a more pronounced decrease in apparent viscosity with increasing shear rate. These trends are consistent with the model coefficients reported in Table 2 and are useful to directly understand how the formulation variables affected the flow behavior of the mixes. In fact, formulations with higher viscosity and consistency generally corresponded to greater flow resistance, in agreement with the positive coefficients estimated for sucrose, inulin, and carob flour. The overall variability covered by the experimental domain can be exploited in the subsequent optimization phase, where the optimal combination of ingredients should be found in order to approach the desired rheological properties while reducing sugar content to reach the nutritional target.

### 3.3. Optimization of Ice Cream Formulation and Validation of the Results

The results obtained within the experimental domain were used to select an optimal formulation according to predefined rheological targets derived from a commercial ice cream. The optimized formulation was experimentally prepared to provide a local assessment of the agreement between predicted and experimental values. Specifically, viscosity, consistency, flow behavior index, and thixotropy of the control and of a commercial reference ice cream were set as target parameters, with an arbitrary tolerance of ±20% (Supplementary Table S6). This tolerance was chosen considering the number of variables involved in the optimization (sugars, inulin, and carob flour) and the technological flexibility required to develop ice creams with a reduced sugar content and potentially high fiber levels. The optimized formulation consisted of the following ingredients (expressed in g, according to the formulation described in Materials and Methods): sucrose 32.06 g, dextrose 8.21 g, inulin 116.45 g, and carob flour 36.27 g.

The comparison between predicted and actual values (Table 4) highlights that during validation, consistency and thixotropy showed relatively good agreement with the model predictions, whereas larger discrepancies were observed for viscosity and flow behavior index. The magnitude of these deviations should also be interpreted considering the wide variability in rheological behavior covered by the experimental domain (Table 3). Notably, consistency and viscosity varied by up to 224-fold and 34-fold, respectively, across the fifteen trials despite the total amount of the four ingredients being held constant. Such a pronounced sensitivity of the rheological response to relatively small compositional changes indicates that achieving a specific, tightly defined rheological target with these functional ingredients is intrinsically challenging. This variability could also explain the relatively low composite desirability obtained during optimization, which reflects the difficulty of reaching all four ideal target values simultaneously. When evaluated against this experimental range, the observed deviation for viscosity, consistency, and thixotropy corresponded to a small fraction of the range spanned by the design (5.2%, 0.2%, and 2.8%, respectively), while the deviation for the flow behavior index represented a considerably larger proportion of its own range (36.5%), suggesting that this parameter was the only one meaningfully outside the model’s predictive capability. Together with the relatively good agreement observed for consistency and thixotropy, these results support the practical usefulness of the models for guiding formulation at the selected point. However, they do not establish robust predictive capability across the entire experimental domain. These discrepancies were expected to some extent, as empirical prediction models are approximations of reality [30], and may not fully capture the complexity of rheological behavior or all interactions among the ingredients. The relatively low composite desirability highlights the difficulty of simultaneously optimizing multiple rheological parameters in a complex food matrix, where improving one response may compromise another. Therefore, the obtained formulation should be regarded as a compromise among the multiple rheological targets, rather than as a formulation reproducing the rheological profile of the commercial reference. To improve the robustness of the optimization, a broader experimental domain and/or additional experimental points could be considered in future work, particularly around the regions close to the target values. This would improve the predictive accuracy of the models. Moreover, further studies should assess other relevant quality attributes, such as melting behavior, texture, microstructure, and storage stability, to provide a more comprehensive evaluation of the reformulated product.

### 3.4. Glucose and Sucrose Contents and the Sweetness Perception of Ice Cream

In addition to the optimization of the rheological properties, the aim of this study was to improve the nutritional properties of ice cream by reducing the glucose and sucrose contents. The concentrations of sucrose and glucose, together with total soluble solids of the ice cream, are depicted in Figure 2. The optimized formulation exhibited significantly lower concentrations of both sucrose and glucose compared not only to the experimental control (formulation no. 5, Table 1) but also to a commercial ice cream used as a benchmark. This result is particularly relevant in light of current nutritional guidelines, which strongly encourage the reduction in added sugars due to their association with metabolic disorders and other health issues [31,32]. In addition to individual sugars, total soluble solids were also significantly lower in the optimized formulation compared to the commercial control. Total soluble solids influence the crystallization phenomena [9] and contribute to the overall structural stability during processing and storage [12]. Despite the reduction observed, the values obtained in this study were within the range typically reported for ice creams in the literature [28,33]. This suggests that the optimized formulation reduced glucose and sucrose while maintaining total solids at an acceptable level, which is essential to achieve the desired rheological properties.

The total dietary fiber content (calculated from nutrition facts) reached 11.59 g/100 g (Table 5). Dietary fibers present in the optimized formulation can be assumed as a mixture of soluble and insoluble fibers derived from inulin and carob flour, respectively [34]. Overall, the increase in fiber content represents a substantial contribution toward EFSA-recommended daily intake of 25 g, established for maintaining normal bowel function and reducing the risk of long-term cardiovascular diseases [35]. Therefore, the optimized formulation could be qualified as a high-fiber product according to Regulation (EC) No 1924/2006, as it more than doubles the minimum requirement of 6 g/100 g established for this claim [36]. Additionally, evaluation of total energy value revealed a relevant reduction, with values decreasing from 222 kcal/100 g in the commercial control to 182.38 kcal/100 g for the experimental control, reaching 158.55 kcal/100 g in the optimized formulation (Table 5).

The sensory analysis was specifically conducted to examine whether sweetness was affected by the sugar substitution by analyzing the differences between the optimal formulation and the experimental control. The commercial control was not included in this test, as the main aim was to identify the changes in sweetness attributable solely to the formulation modification, i.e., within the experimental domain using the same food matrix. In particular, Figure 3 shows the percentage variation in perceived sweetness of the optimized sample relative to the control, with a median decrease of −25%. The statistical analysis (paired *t*-test) confirmed that this difference was significant (*p* = 0.006), indicating that the reduction in sucrose and glucose contents (approximately 71% according to Table 5) led to a measurable decrease in perceived sweetness. This effect can be directly related to the lower concentration of glucose and sucrose, which are the primary contributors to sweetness perception. Nevertheless, the contribution of carob-derived sensory characteristics cannot be completely excluded. Although the statistical significance indicates that trained assessors were able to discriminate between samples, such differences may not always directly translate into reduced consumer acceptance. In this regard, further studies involving untrained panelists or consumer tests would be necessary to assess the real perceptibility of sweetness reduction and its impact on hedonic response. Overall, the results could be considered a compromise to reach the desired the nutritional profile.

## 4. Conclusions

This study demonstrated that the rheological properties of ice cream can be effectively modulated through a rational combination of sucrose, dextrose, inulin, and carob flour, achieving the reformulation with reduced glucose and sucrose while maintaining broadly comparable rheological properties. The mixture design approach used in this study allowed us to understand the effects of each ingredient on the rheological properties of ice cream. An optimal formulation was subsequently identified and experimentally validated. Although significant differences from the commercial reference remained for all rheological parameters, the optimized formulation provided a compromise among the multiple rheological targets considered. Overall, the experimental design strategy provided a useful approach for formulation development, although further refinement of the predictive models is warranted.

In addition to the technological results, the optimized formulation showed a marked improvement in nutritional quality, with a significant reduction in sucrose and glucose and a lower energy value than the control. The total dietary fiber content was also high enough to potentially support the claim “high in fiber” under current European legislation. However, the optimized formulation showed a significantly lower perceived sweetness than the experimental control. Although this reduction was associated with a substantial decrease in simple sugars, the contribution of carob-derived sensory characteristics cannot be completely excluded. Therefore, lower sweetness may represent an important factor influencing consumer acceptance and purchase intention and should be investigated through dedicated consumer studies. Future studies should further refine the predictive models by expanding the experimental domain and including additional formulation and processing variables. Finally, consumer studies are also needed to determine whether the moderate reduction in perceived sweetness observed is acceptable to consumers and how it affects overall product liking and purchase intention.

## Figures and Tables

**Figure 1:**
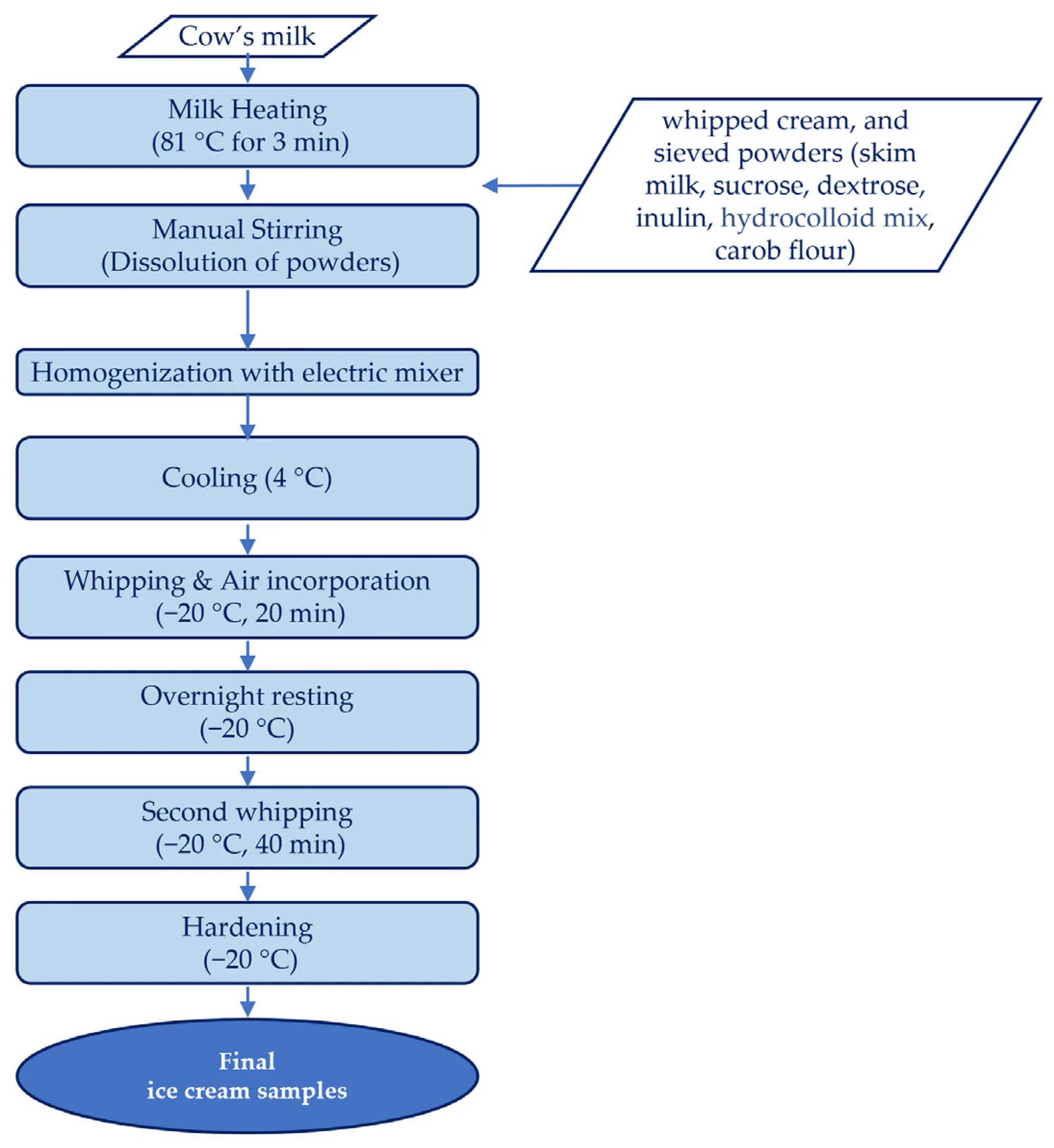
Flow diagram of ice cream making.

**Figure 2:**
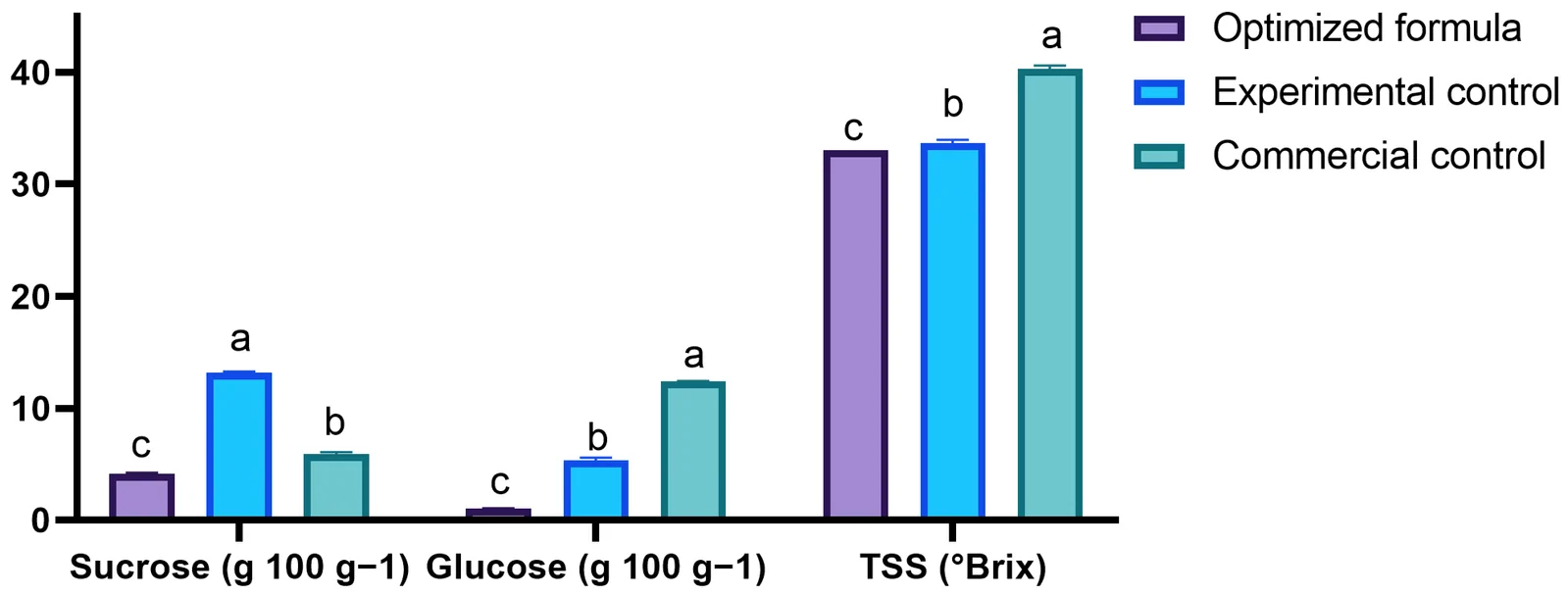
Sucrose (g 100 g^−1^), glucose (g 100 g^−1^), and total soluble solids (TSS) of ice creams. Data are expressed as a mean ± SD (n = 3). Different letters for the same parameter indicate significant differences according to Tukey’s HSD test (*p* ≤ 0.05).

**Figure 3:**
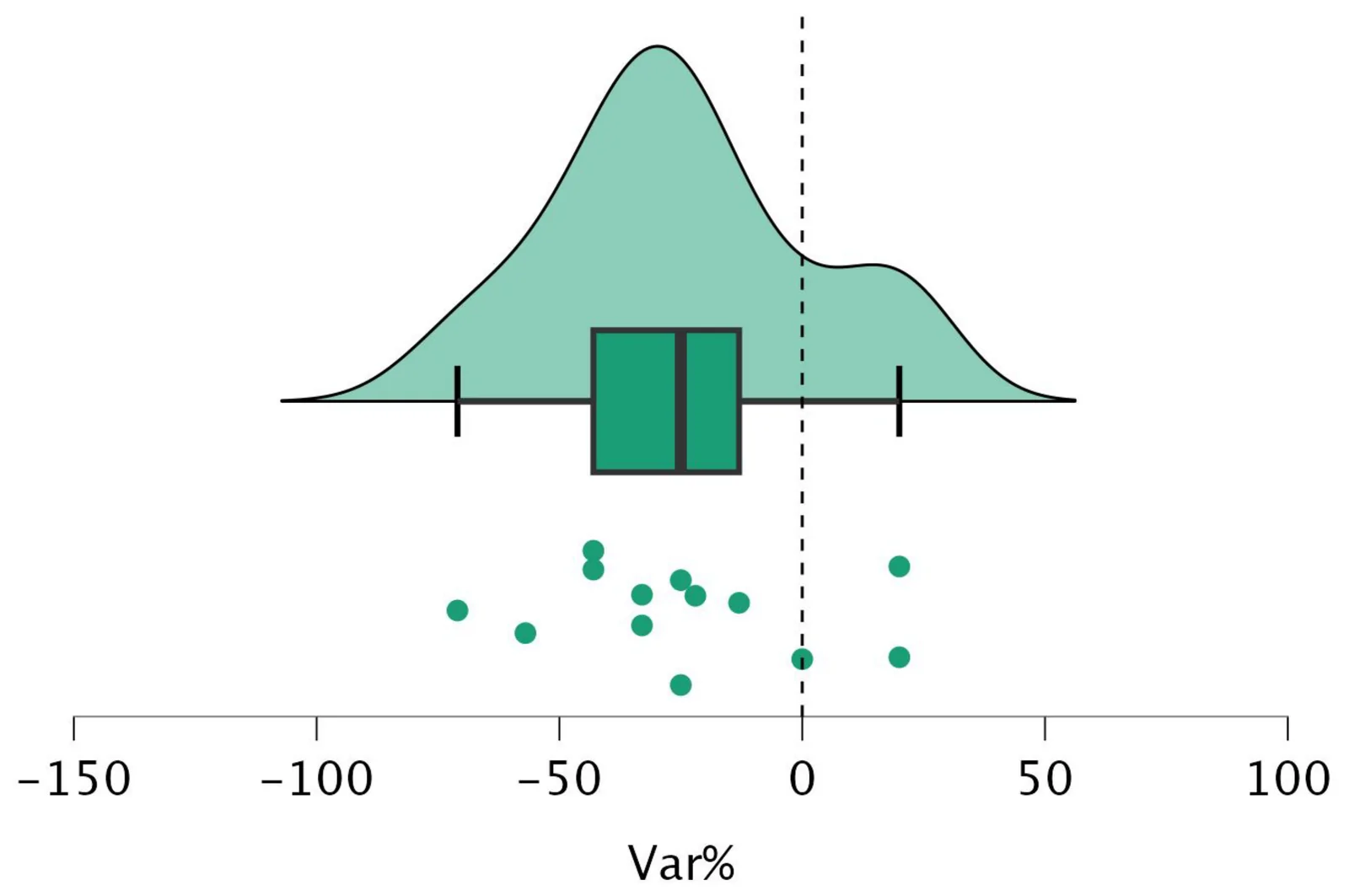
Frequency distribution, box plot (the bold line corresponds to the median), and single points of the percentage variation in perceived sweetness of the optimized sample relative to the control.

**Table 1:** Composition of ice cream formulations according to the Design of Experiment. Data expressed as g kg^−1^.

Trial	Sucrose (g)	Dextrose (g)	Inulin (g)	Carob Flour (g)
1	0	0	193	0
2	0	0	0	193
3	154	0	39	0
4	110	8.36	47.07	27.57
5 *	154	39	0	0
6	33	8.36	124.07	27.57
7	33	27.86	27.57	104.57
8	0	39	154	0
9	110	8.36	27.57	47.07
10	33	8.36	27.57	124.07
11	0	39	0	154
12	110	27.86	27.57	27.57
13	66	16.71	55.14	55.14
14	154	0	0	39
15	33	27.86	104.57	27.57

* Indicates that formulation no. 5 is the control, with no added inulin or carob flour.

**Table 2:** Estimated regression coefficients of the models and their significance.

Term	Viscosity at 50 s^−1^ (η, Pa s)	Consistency (K, Pa s^n^)	n	Thixotropy (%)
Sucrose	**8.9**	**146**	**0.500**	**14.7**
Dextrose	**−680.2**	**−13,143**	**7.879**	**216.4**
Inulin	**5**	**158**	**0.124**	**29.4**
Carob Flour	**8.9**	**108**	**0.388**	**31.4**
Sucrose × Dextrose	809.7	**15,736**	−7.842	−283.8
Sucrose × Inulin	−44.7	**−906**	1.057	8.4
Sucrose × Carob Flour	−48.9	**−804**	0.937	−27.9
Dextrose × Inulin	836.3	**15,796**	−8.416	−281.8
Dextrose × Carob Flour	819.9	**16,018**	−8.499	−308.1
Inulin × Carob Flour	**−39**	**−940**	0.914	4.9
R^2^	**96.09**	**97.92**	**95.17**	**92.99**
R^2^_adj_	**89.05**	**94.17**	**86.49**	**80.39**
*p*-Value	**<0.01**	**<0.01**	**<0.01**	**0.02**
R^2^ Predicted (PRESS-based)	−109.55	−15.61	−127.93	−207.41
Adequate Precision	14.06	17.64	12.45	9.53

Values in bold indicate the significant coefficients (*p* ≤ 0.05). R^2^_adj_—adjusted coefficient of determination; PRESS—prediction residual error sum of squares.

**Table 3:** Rheological parameters of the experimental ice creams.

Trial	Viscosity at 50 s^−1^ (η, Pa s)	Consistency (K, Pa s^n^)	n	% Thixotropy
1	4.96 ± 0.22	161.35 ± 10.11	0.121 ± 0.018	30.39 ± 0.17
2	9.15 ± 0.93	108.55 ± 8.20	0.371 ± 0.043	30.35 ± 9.23
3	0.48 ± 0.02	2.24 ± 0.15	0.609 ± 0.020	20.24 ± 0.81
4	1.00 ± 0.01	5.14 ± 0.30	0.582 ± 0.012	17.55 ± 1.68
5	0.43 ± 0.04	1.36 ± 0.30	0.708 ± 0.032	9.81 ± 1.07
6	1.13 ± 0.04	9.99 ± 0.96	0.453 ± 0.016	20.35 ± 1.47
7	1.96 ± 0.13	11.7 ± 1.27	0.543 ± 0.011	15.40 ± 0.73
8	1.15 ± 0.04	16.39 ± 2.09	0.333 ± 0.024	21.66 ± 2.03
9	0.86 ± 0.40	3.43 ± 1.67	0.647 ± 0.006	14.65 ± 1.51
10	1.57 ± 0.01	6.73 ± 0.09	0.624 ± 0.001	25.07 ± 0.05
11	1.84 ± 0.05	10.90 ± 0.88	0.545 ± 0.023	18.87 ± 1.78
12	0.27 ± 0.00	0.72 ± 0.03	0.754 ± 0.006	11.01 ± 0.78
13	0.76 ± 0.11	4.02 ± 1.23	0.586 ± 0.043	15.37 ± 1.65
14	1.18 ± 0.28	5.17 ± 1.75	0.627 ± 0.036	12.16 ± 2.76
15	1.79 ± 0.06	12.09 ± 0.85	0.516 ± 0.010	20.87 ± 1.07

Mean ± SD (n = 3).

**Table 4:** Rheological parameters of the optimized formulation and commercial ice creams.

	Optimized Formula (Predicted Values)	Optimized Formula (Actual Values)	Commercial Ice Cream
Viscosity at 50 s^−1^ (η, Pa s)	0.53	0.99 ± 0.02 a	0.45 ± 0.00 b
Consistency (K, Pa s^n^)	2.91	3.20 ± 0.10 a	2.46 ± 0.10 b
n	0.47	0.70 ± 0.00 a	0.56 ± 0.01 b
Thixotropy (%)	24.08	23.497 ± 0.31 b	28.22 ± 3.58 a
Composite desirability	0.064		

Data are expressed as a mean ± SD (n = 3). Different letters in the same row indicate significant differences according to Tukey’s HSD test (*p* ≤ 0.05).

**Table 5:** Total dietary fibers and energy value of the ice cream formulations.

	Total Dietary Fibers (g 100 g^−1^)	Sum of Glucose and Sucrose (g 100 g^−1^)	Glucose and Sucrose Reduction Achieved in Optimized Formulation with Respect to Controls (%)	Energy Value (kcal 100 g^−1^)
Optimized formulation	11.59	5.21 ± 0.10	-	158.55
Experimental control	-	18.54 ± 0.27	−71.89%	182.38
Commercial control	-	18.35 ± 0.17	−71.59%	222

Total dietary fibers and energy values are calculated according to the nutritional facts of the ingredients, considering the contents of protein, fat, lipids, and dietary fibers.

## Data Availability

The original contributions presented in this study are included in the article/Supplementary Materials. Further inquiries can be directed to the corresponding author.

## Supplementary Materials

The following supporting information can be downloaded at: https://www.mdpi.com/article/10.3390/foods15193362/s1, Table S1: Model fitting parameters (Chi-squared, r) for the Ostwald-de Waele model applied to the experimental flow curves of the ice cream trials; Table S2: Analysis of variance (ANOVA) for the fitted model describing the viscosity of the ice cream formulations; Table S3: Analysis of variance (ANOVA) for the fitted model describing the consistency index of the ice cream formulations; Table S4: Analysis of variance (ANOVA) for the fitted model describing the flow behavior index of the ice cream formulations; Table S5: Analysis of variance (ANOVA) for the fitted model describing the thixotropy index of the ice cream formulations; Table S6: Target values and tolerance for the optimization of ice cream formulation; Figure S1: Residual diagnostics for the four response surface models (viscosity, consistency, flow behavior index, thixotropy). Left: residuals vs. predicted values; right: normal Q-Q plot of internally studentized residuals.
