# Supplementary material for: Rheological Optimization of Reduced-Sugar Ice Cream Enriched with Inulin and Carob Flour
Source: Foods. 2026 Sep 22;15(19):3362. doi: 10.3390/foods15193362 (PMC13664442; doi:10.3390/foods15193362)
Supplement: Supplementary file 1 [file foods-15-03362-s001.zip › foods-4501820-supplementary.pdf]

Table S1 Model fitting parameters (Chi-squared, r) for the Ostwald-de Waele model applied to the experimental flow curves of the ice cream trials.

Trial	Chi-squared	r
1	17.00 ± 7.91	0.993 ± 0.002
2	4.06 ± 4.08	0.999 ± 0.001
3	0.02 ± 0.01	0.994 ± 0.002
4	0.01 ± 0.00	0.999 ± 0.000
5	0.00 ± 0.00	0.997 ± 0.000
6	0.22 ± 0.02	0.994 ± 0.000
7	0.01 ± 0.00	1.000 ± 0.000
8	0.51 ± 0.14	0.992 ± 0.001
9	0.02 ± 0.03	0.994 ± 0.008
10	0.04 ± 0.00	0.999 ± 0.001
11	0.03 ± 0.02	1.000 ± 0.000
12	0.00 ± 0.00	0.998 ± 0.000
13	0.02 ± 0.01	0.999 ± 0.000
14	0.01 ± 0.01	0.999 ± 0.000
15	0.11 ± 0.01	0.999 ± 0.000

Mean ± SD

Table S2. Analysis of variance (ANOVA) for the fitted model describing the viscosity of the ice cream formulations.

Source	DF	Seq SS	Adj SS	Adj MS	F	P
Regression	9	70.92	70.92	7.88	13.65	0.01
Linear	3	44.71	22.46	7.49	12.97	0.01
Quadratic	6	26.21	26.21	4.37	7.57	0.02
Sucrose*Dextrose	1	7.37	3.11	3.11	5.39	0.07
Sucrose*Inulin	1	3.32	2.69	2.69	4.66	0.08
Sucrose*Carob	1	7.16	3.23	3.23	5.60	0.06
Dextrose*Inulin	1	2.29	2.98	2.98	5.16	0.07
Dextrose*Carob	1	0.07	2.86	2.86	4.96	0.08
Inulin*Carob	1	5.99	5.99	5.99	10.38	0.02
Residual Error	5	2.89	2.89	0.58		
Total	14	73.80				

Table S3. Analysis of variance (ANOVA) for the fitted model describing the consistency index of the ice cream formulations.

Source	DF	Seq SS	Adj SS	Adj MS	F	P
Regression	9	29465.30	29465.30	3273.92	26.12	0.00
Linear	3	15687.90	8195.50	2731.82	21.79	0.00
Quadratic	6	13777.40	13777.40	2296.23	18.32	0.00
Sucrose*Dextrose	1	4317.00	1176.10	1176.06	9.38	0.03
Sucrose*Inulin	1	3319.10	1107.40	1107.43	8.83	0.03
Sucrose*Carob	1	2219.80	873.00	873.00	6.96	0.05
Dextrose*Inulin	1	420.40	1061.80	1061.78	8.47	0.03
Dextrose*Carob	1	13.20	1091.80	1091.82	8.71	0.03
Inulin*Carob	1	3488.00	3488.00	3488.03	27.83	0.00
Residual Error	5	626.80	626.80	125.35		
Total	14	30092.10				

Table S4. Analysis of variance (ANOVA) for the fitted model describing the flow behavior index of the ice cream formulations.

Source	DF	Seq SS	Adj SS	Adj MS	F	P
Regression	9	0.35	0.35	0.04	10.96	0.01
Linear	3	0.29	0.06	0.02	5.87	0.04
Quadratic	6	0.05	0.05	0.01	2.45	0.17
Sucrose*Dextrose	1	0.00	0.00	0.00	0.08	0.78
Sucrose*Inulin	1	0.02	0.00	0.00	0.43	0.54
Sucrose*Carob	1	0.02	0.00	0.00	0.34	0.59
Dextrose*Inulin	1	0.00	0.00	0.00	0.09	0.78
Dextrose*Carob	1	0.00	0.00	0.00	0.09	0.78
Inulin*Carob	1	0.00	0.00	0.00	0.94	0.38
Residual Error	5	0.02	0.02	0.00		
Total	14	0.36				

Table S5. Analysis of variance (ANOVA) for the fitted model describing the thixotropy index of the ice cream formulations.

Source	DF	Seq SS	Adj SS	Adj MS	F	P
Regression	9	512.74	512.74	56.97	7.38	0.02
Linear	3	444.26	37.31	12.44	1.61	0.30
Quadratic	6	68.48	68.48	11.41	1.48	0.34
Sucrose*Dextrose	1	2.57	0.38	0.38	0.05	0.83
Sucrose*Inulin	1	0.48	0.10	0.10	0.01	0.92
Sucrose*Carob	1	59.22	1.05	1.05	0.14	0.73
Dextrose*Inulin	1	5.81	0.34	0.34	0.04	0.84
Dextrose*Carob	1	0.32	0.40	0.40	0.05	0.83
Inulin*Carob	1	0.09	0.09	0.09	0.01	0.92
Residual Error	5	38.62	38.62	7.72		
Total	14	551.36				

Table S6. Target values and tolerance for the optimization of ice cream formulation.

	Goal	Lower	Target	Upper	Weight	Import
Viscosity at 50 s^{-1} (η, Pa s)	Target	0.36	0.45	0.54	1	1
% Thixotropy	Target	24	30	36	1	1
Consistency (K, Pa s^n)	Target	1.97	2.46	2.95	1	1
n	Target	0.45	0.56	0.67	1	1

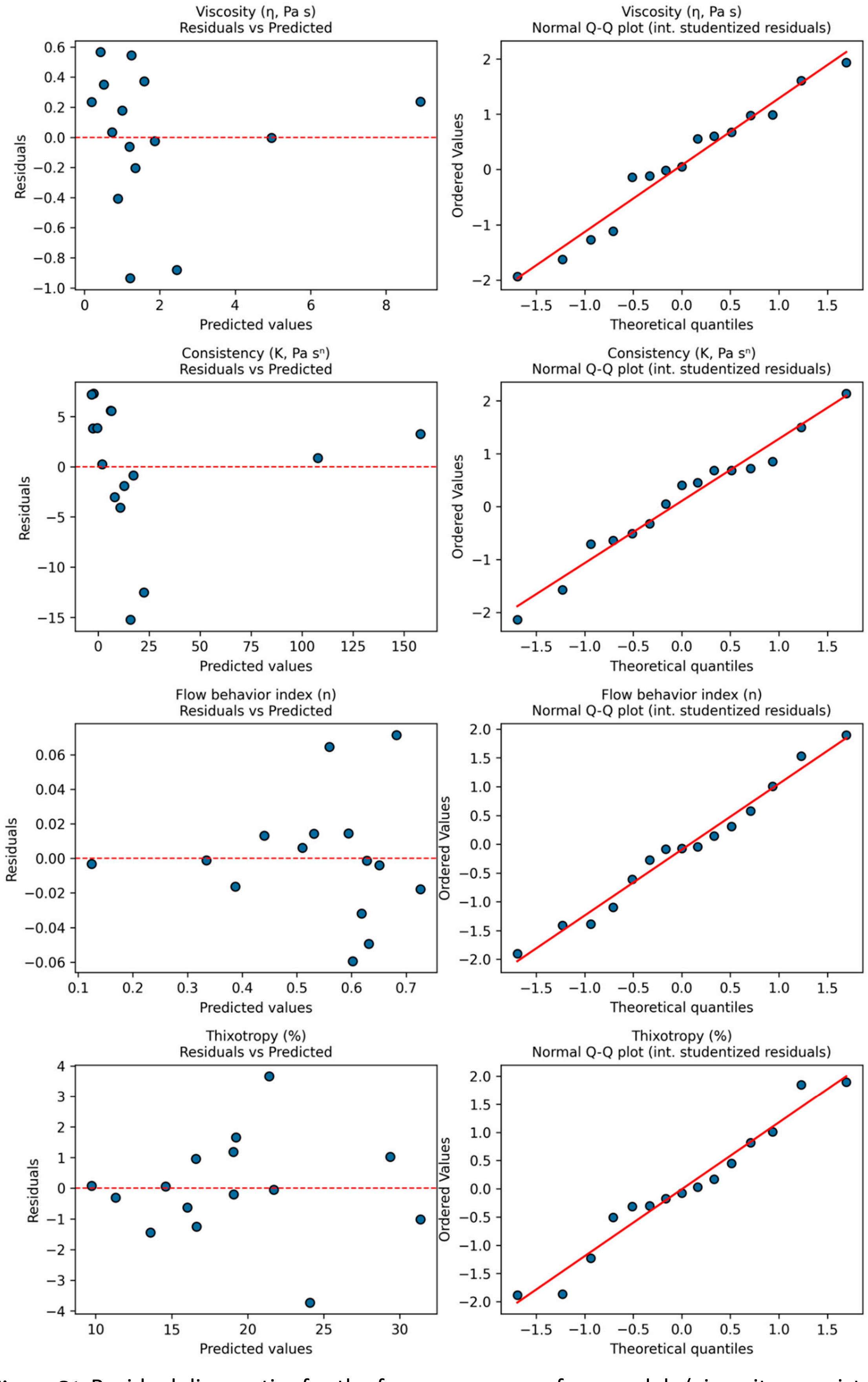

Figure S1. Residual diagnostics for the four response surface models (viscosity, consistency, flow behavior index, thixotropy). Left: residuals vs. predicted values; right: normal Q-Q plot of internally studentized residuals.
